# Evaluation of barium–strontium nanoferrite-based sensors for VOC detection: the case of ethanol and acetone

**DOI:** 10.1039/d6ra02708c

**Published:** 2026-06-04

**Authors:** B. Abdellaoui, A. Benali, M. Bejar, E. Dhahri, J. Wu

**Affiliations:** a Laboratoire de Physique Appliquée, Faculté des Sciences, Université de Sfax B. P. 1171 3000 Sfax Tunisia boutheinaabdelloui@gmail.com +216-74676609 +216-96162555; b I3N and Physics Department, University of Aveiro Aveiro 3810-193 Portugal; c Faculté des Sciences de Monastir, Universit, é, de Monastir Tunisia; d College of Chemistry and Materials Science, Sichuan Normal University Chengdu 610068 China

## Abstract

Over the past few decades, traditional approaches to detecting volatile organic compounds (VOCs) have been transformed by the integration of data intelligence, allowing valuable insights into sensor behavior when exposed to different gases. In gas sensing, VOCs such as acetone and ethanol are commonly used to evaluate sensors due to their closely related chemical properties, which makes distinguishing between them particularly challenging. In this study, we evaluated the detection of ethanol and acetone gases using spinel nanoferrites Ba_0.5−*x*_Sr_*x*_Fe_2.5_O_4_ (*x* = 0.00, 0.25 and 0.50). The materials exhibit structural features that favor gas adsorption and surface reactivity. Electrical measurements confirmed their n-type semiconductor behavior, and sensing tests performed over a wide range of gas concentrations (500 ppb to 100 ppm) demonstrated high sensitivity, even at very low concentrations. All three compositions showed exceptionally low detection limits, which represents a key advantage for the rapid and efficient detection of acetone and ethanol. Furthermore, the sensors displayed high sensitivity, exceeding values previously reported in the literature, along with very short response and recovery times, highlighting their strong potential for real-time applications. Our results indicate that Ba^2^/Sr^2^ substitution does not significantly affect VOC sensitivity, but emphasize the crucial role of the nanostructured architecture of the ferrites in enhancing gas sensing performance.

## Abstract 2

The synthesized nanostructured materials were integrated into a gas sensor, which showed superior response and sensitivity toward target gases, indicating its suitability for environmental monitoring applications.

## Graphical abstract

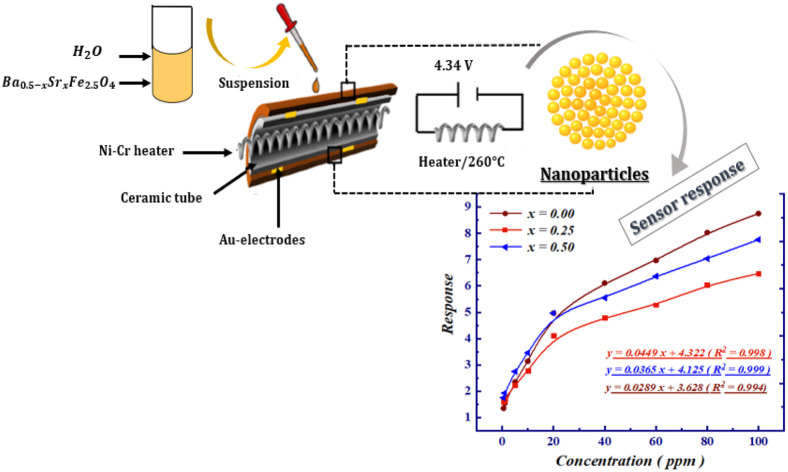

## 1 Introduction

Gas sensors play a crucial role in industrial and environmental applications, enabling real-time monitoring of potentially hazardous gaseous molecules. Their ability to detect low gas concentrations contributes to the safety of industrial processes, the prevention of explosion and poisoning risks, and the control of air pollution.^1–5^ Acetone ((CH_3_)_2_CO), widely used in various industrial processes,^6^ poses health risks.^7^ It also serves as a biomarker for diabetes, with concentrations ranging from 0.3–0.9 ppm in healthy individuals to 1.8 ppm in type II diabetic patients.^8^ Ethanol, used as a solvent, biofuel, disinfectant, or component of alcoholic beverages,^9^ is also a major safety concern, being involved in numerous fatal accidents.^10^ Its detection requires highly sensitive (<10 ppm) and stable sensors, as physiological effects appear at concentrations between 250 and 1000 ppm.^11^

Current research aimed at improving the performance of gas sensors operating at room temperature (RT) is primarily focused on optimizing the intrinsic properties of sensing materials as well as developing external modulation strategies, among which atomic defect engineering plays a central role. These defects are crucial as they create active sites that promote gas molecule chemisorption^12–14^ and modulate surface electronic states, thereby enhancing adsorption processes and charge transfer kinetics,^15–17^ ultimately leading to improved sensing performance.^18,19^

In addition, light-assisted modulation has emerged as an effective approach to enhance RT gas-sensing performance by precisely controlling surface charge transfer processes and defect-influenced adsorption energetics, thereby simultaneously improving sensitivity and response/recovery stability.^20–22^ Several studies have also demonstrated that photoinduced charge carrier dynamics, involving electron–hole pair separation, can trigger oxygen desorption from the surface and lead to the formation of oxygen vacancies through interfacial redox processes.^23,24^

In this context, *operando* studies have highlighted the critical role of oxygen vacancies in sensing mechanisms, particularly in CeO_2_ and SnO_2_ under exposure to different gases.^25,26^ Similarly, Junker *et al.*^27^ demonstrated *via* DRIFTS spectroscopy that oxygen photodesorption at the WO_3_ surface is a key process in sensing under heating conditions, although this mechanism is not directly applicable to room-temperature operation. However, due to the complexity of gas–solid interactions under photoexcitation, the dynamics of oxygen vacancies at RT remain insufficiently understood.

Furthermore, Ji Li *et al.*^28^ investigated the photoinduced generation of oxygen vacancies in In_2_O_3_ and their impact on NO_2_ detection at room temperature. Their results, obtained through *operando* Raman and *in situ* DRIFTS analyses, showed that oxygen vacancies enhance the formation of adsorbed oxygen species, promote surface charge transfer, and act as active sites for NO_2_ chemisorption and redox reactions. These findings emphasize the importance of photoinduced defect engineering as a promising strategy for developing high-performance optoelectronic gas sensors operating at room temperature.

More recently, Xinchao Li *et al.*^29^ further confirmed the growing interest in high-entropy oxides (HEOs) for gas-sensing applications. In this context, FeCoNiCrMn high-entropy alloys were used as precursors to synthesize the spinel high-entropy oxide (FeCoNiCrMn)_3_O_4_. The obtained material was systematically investigated in terms of crystal structure, morphology, elemental valence states, and gas-sensing performance. Notably, the sensor exhibited a strong response to NO_2_ at room temperature without any surface modification or sensitization treatment, highlighting the potential of high-entropy oxide systems for advanced gas-sensing applications.

n-type MOS are distinguished by their wide bandgap, good thermal stability, and high gas sensitivity, making them particularly suitable materials for the development of reliable and high-performance sensors.

Within this family, semiconductor ferrites (MFe_2_O_4_, with M = Ba, Sr, Ni, Co, *etc*.) occupy a special place. These spinel oxides contain mixed-valence ions, which enable charge transport through small polaron hopping mechanisms.

This property makes the electrical conductivity of ferrites highly sensitive to the adsorption of gas molecules, unlike metals, allowing precise and selective detection. Several nanoferrites have demonstrated remarkable performance: BaFe_2_O_4_ shows excellent sensitivity to acetone and ethanol (0.5–100 ppm) with strong selectivity at 100 ppm;^30^ Co_3_O_4_ ferrites deliver comparable results;^31^ and ZnFe_2_O_4_ and CdFe_2_O_4_ nanoparticles achieve maximum response to 100 ppm of acetone and ethanol at 250 °C.^32^ Spinel ferrites, due to their electrical, magnetic, and chemical properties, are ideal for detecting toxic gases at low concentrations from industrial, automotive, and environmental sources.^33^ Their thermal stability, tunable resistivity, and strong reactivity toward reducing gases make them particularly suitable for resistive-type sensors.^34^ The choice of the cation M in MFe_2_O_4_ strongly influences the crystal structure, Fe–O bonding, charge carrier mobility, and microstructure.^35^ In particular, the progressive substitution of Ba^2^ with Sr^2+^ (smaller ionic radius: 1.18 Å *vs.* 1.35 Å) modifies porosity, crystallite size, and defect density, optimizing gas adsorption and charge transport.^36,37^ However, an excessive increase of Sr does not necessarily lead to a linear improvement in response, as the structure and microstructure reach an optimal balance of active sites.

Based on these considerations, the composition Ba_0.5−*x*_Sr_*x*_Fe_2.5_O_4_ was selected in this study to exploit the redox properties of iron while optimizing the crystal structure and microstructure through cationic substitution. The results demonstrate superior performance for acetone and ethanol detection, with rapid response and recovery times and high sensitivity, confirming the potential of these materials for developing next-generation sensors suitable for monitoring gaseous biomarkers.

## 2 Experimental methods

### 2.1 Compounds preparation

**Fig. 1:**
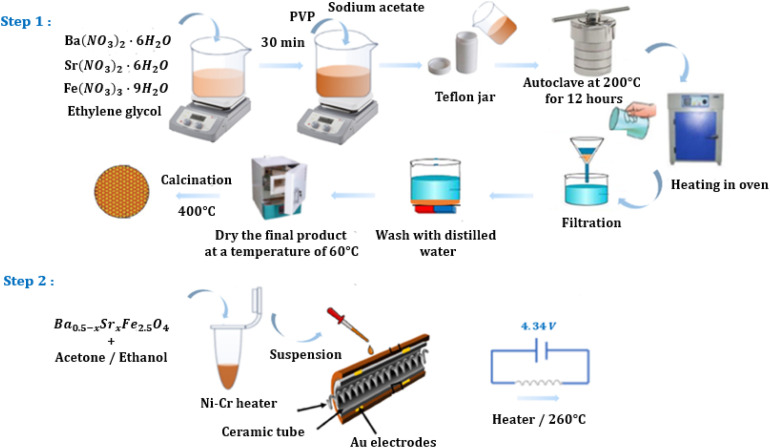
(Steps 1 and 2): Schematic diagram illustrating the preparation process of gas sensors based on Ba_0.5−*x*_Sr_*x*_Fe_2.5_O_4_ (*x* = 0.00, 0.25 and 0.50) compounds.

The compounds with the formula Ba_0.5−*x*_Sr_*x*_Fe_2.5_O_4_(*x* = 0.00; 0.25 and 0.50) were synthesized using the solvothermal method. This technique was selected for its ability to promote inorganic polymerization reactions at near-room temperature, enabling the formation of powders with a well-controlled structure. This synthesis approach contributes to the development of gas sensors based on spinel-type metal oxides. All the synthesis steps were detailed in our previous work,^38^ and summarized in Fig. 1 (step 1).

### 2.2 Gas sensors preparation

The Ba_0.5−*x*_Sr_*x*_Fe_2.5_O_4_(*x* = 0.00, 0.25 and 0.50) compounds were used in powder form for the fabrication of thick-film gas sensors. After homogeneous dispersion in distilled water, the powders were transformed into a viscous paste suitable for deposition.

The resulting suspension was then dropwise deposited onto ceramic tubes (4 mm length, 1.2 mm diameter) equipped with gold (Au) electrodes to form the sensitive layer of the sensor. A nickel–chromium (Ni–Cr) alloy heating wire was inserted through the tubes to ensure precise control of the operating temperature.

Then, the operating temperature of the sensor was set to 260 °C for 48 hours to improve its stability (heating voltage at 4.34 V).

Subsequently, the sensor's detection performance was characterized using a WS-30A system (Zhengzhou Weisen Electronics Technology, China) (Fig. 1 (step 2)). The target gas, an analytical grade (>99%) volatile organic compound (VOC) liquid, was collected using a pipette and then deposited onto a built-in heating element inside the test chamber. This liquid was instantly vaporized into the ambient air, creating a homogeneous gas/air mixture maintained at a constant temperature of 25 °C. A fan, placed near the heating element, was used to promote forced convection, ensuring good distribution of the vapor throughout the volume of the chamber.

The volume *V* of the VOC liquid required to obtain a given gas concentration can be estimated using the following eqn (1):

1 *V*(m^3^) = [*M*(kg mol^−1^) × *P*^*θ*^(Pa) × *L*(m^3^) × *C*(ppm)]/[*ρ*(kg m^−3^) × *ω* × *R*(P_a_ m^3^ mol^−1^ K^−1^) × *T*(K)]

where *M* represents the molar mass, *P*^*θ*^ the standard atmospheric pressure, *L* the volume of the test chamber (0.0173 m^3^), *C* the mole fraction corresponding to the target gas concentration (1 ppm = 1 × 10^−6^), *ρ* the density of the VOC liquid/solution, *ω* the mass percentage of the VOC (for a pure liquid, *ω* = 1; for a formaldehyde (HCHO) solution, *ω* = 0.37), *R* the ideal gas constant (8.315 *P*_a_ m^3^ mol^−1^ K^−1^), and *T* the temperature expressed in kelvins. The water vapor produced by the VOC solution can be considered negligible.

The gas response (*S*) is determined using the formula *S* = *R*_a_/*R*_g_,^39^ where *R*_a_and *R*_g_ represent the resistance in ambient air and in the vapor of the target volatile organic compound (VOC), respectively.

## 3 Results and discussion

### 3.1 Structural characterization

**Fig. 2:**
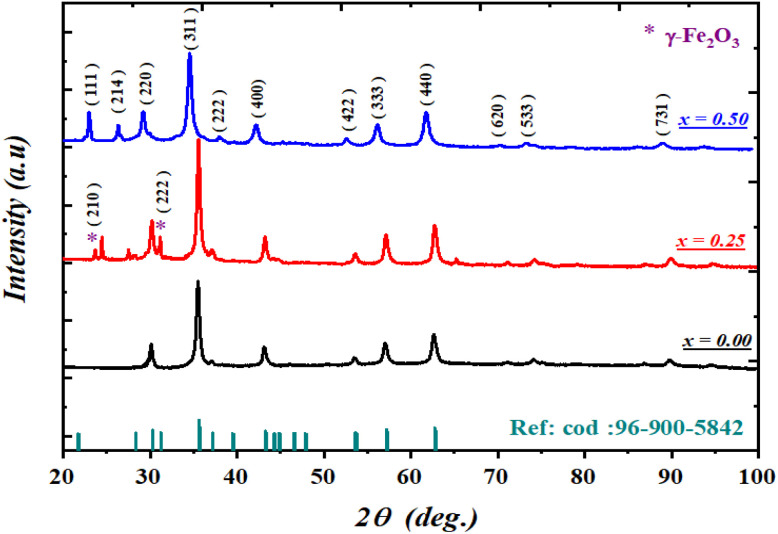
X-ray diffraction (XRD) patterns of the samples with *x* = 0.00, 0.25, and 0.50, along with a comparison between the experimental diffraction data and the standard JCPDS (PDF) card No. 96-900-5842.

The synthesized compounds were structurally characterized by X-ray diffraction (XRD) using a diffractometer equipped with a Cu Kα radiation source (λ = 1.5406 Å)and a graphite monochromator. The powder diffraction patterns were recorded at room temperature over a 2*θ* range of 20°–100° with a scanning step of 0.02°. The experimental diffractograms were indexed and compared with the standard JCPDS (PDF) card No. 96-900-5842, corresponding to Ba_0.5−*x*_Sr_*x*_Fe_2.5_O_4_ with a cubic spinel structure (Fig. 2). The Rietveld refinement of the XRD data was carried out using the FullProf program.

The refinement results confirm that all Ba_0.5−*x*_Sr_*x*_Fe_2.5_O_4_ compounds (*x* = 0.00, 0.25, 0.50) crystallize in a cubic spinel structure belonging to the *Fd*-3̄*m* space group (Fig. 2). The corresponding refinement patterns were previously reported and discussed in detail in our earlier work,^40^ while the main structural parameters obtained from the refinement are summarized in Table 1.

**Table 1:** Summary of the structural parameters obtained from X-ray diffraction (XRD) analysis for the Ba_0.5−*x*_Sr_*x*_Fe_2.5_O_4_ compounds

*x*	0.00	0.25	0.50
*a* (Å)	8.380	8.364	8.358
*V* (Å^3^)	588.616	585.218	583.921
*χ* ^2^	1.02	1.29	2.57
*R*_wp_ (*%*)	1.11	1.16	1.57
*D*_SC_ (nm)	17.905	14.450	12.267
*D*_MEB_ (µm)	0.821	0.601	0.479
*D*_MEB_ (µm)/*D*_SC_ (nm)	45.860	41.591	39.050
*ε* (×10^−3^)	1.345	1.366	3.153
*D*_W−H_ (nm)	52.920	32.000	30.900
*δ* (×10^−4^)	3.571	9.765	10.473
*ρ*_The_ (g cm^−3^)	6.146	5.899	5.628
*ρ*_exp_ (g cm^−3^)	4.240	4.240	4.240
*P* (%)	31.01	28.12	24.67
*S* (m^2^ g^−1^)	79.033	97.930	115.357

The nearly identical diffraction peak positions observed for all compositions indicate the absence of any significant phase transition and confirm the structural stability of the spinel phase upon Sr substitution. However, for the sample with *x* = 0.25, a very weak additional diffraction peak is detected, suggesting the presence of a minor secondary phase identified as γ-Fe_2_O_3_, formed during the synthesis process. The impurity phase content is estimated to be below 4%, indicating that the sample remains predominantly single-phase and that the main crystal structure is not significantly affected.

Furthermore, the low reliability factors *R*_WP_ (1.11–1.57%) and goodness-of-fit values *χ*^2^ (1.02–2.57) demonstrate an excellent agreement between the experimental and calculated diffraction patterns, confirming the high quality and reliability of the Rietveld refinement.

A gradual decrease in both the lattice parameter (*a*)and the unit-cell volume (*V*)is observed with increasing Sr substitution. This lattice contraction can be attributed to the reduction in the average ionic radius at the A-site 〈*r*_*A*_〉, as expressed by eqn (2):

2 〈*r*_A_〉 = (0.5 − *x*)*r*_Ba_^2+^ + *xr*_Sr_^2+^

where *r*_Ba_^2+^ = 1.35 Åand *r*_Sr_^2+^ = 1.18 Å.^40^ The decrease in the lattice parameters confirms the successful incorporation of the smaller Sr^2+^ ions into the spinel lattice, leading to a contraction of the crystal structure.

The average crystallite size (*D*_SC_)was estimated from the XRD patterns using the Debye–Scherrer equation:^40^3
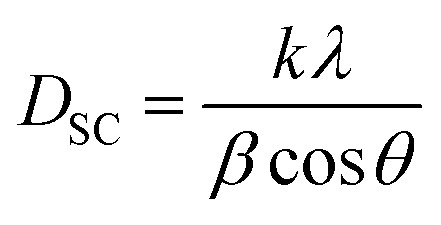
where *θ* is the diffraction angle of the most intense peak, *β* is the full width at half maximum (FWHM), *k* = 0.9 is the shape factor assuming spherical crystallites, and *λ*is the X-ray wavelength.

The calculated *D*_SC_ values, summarized in Table 1, indicate nanometric crystallite sizes for all investigated samples, with a noticeable decrease as the Sr concentration increases. This reduction is closely associated with the contraction of the lattice parameters and suggests that Sr substitution suppresses crystallite growth.

Taking into account the anisotropy of the material properties with respect to the considered crystallographic direction, the microstrain induced by lattice imperfections and crystalline distortions in the powders can be expressed by the following relation:^41^4
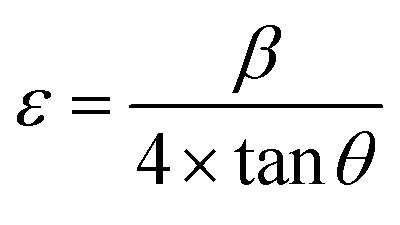
where *β* represents the broadening of the diffraction peak and *θ* the diffraction angle.

Furthermore, the broadening of the diffraction peaks mainly results from two independent contributions: the reduction in crystallite size and the presence of lattice strain within the crystal structure.

Such nanosized crystallites are particularly advantageous for gas sensing applications, as they provide a larger effective surface area, a higher density of active adsorption sites, and enhanced gas–surface interactions, thereby improving the sensing performance.^42,43^

The X-ray density (*ρ*_X-ray_) was calculated taking into consideration that a basic unit cell of the cubic spinel structure contained eight ions, according to the following formula:^44^5
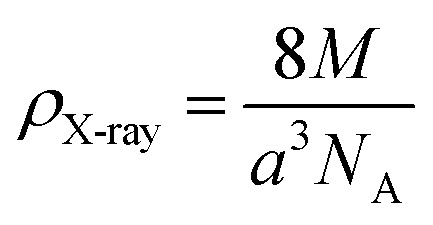
where *Z* = 8 represents the number of formula units per unit cell of the spinel lattice, *M*is the molecular weight of the compound, *a* is the calculated lattice parameter expressed in centimeters, and *N*_A_ is Avogadro's number.

The bulk density *ρ*_exp_ was estimated using the following relation:^44^6
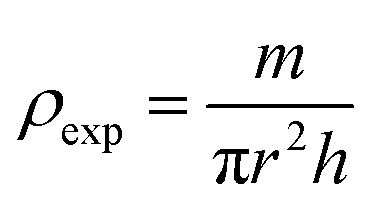
where *h*, *r*, and *m* represent, respectively, the thickness, radius, and mass of the pellet.

The concept of porosity was determined by considering the difference between the X-ray density and the experimental density, formulated as follows:^40^7
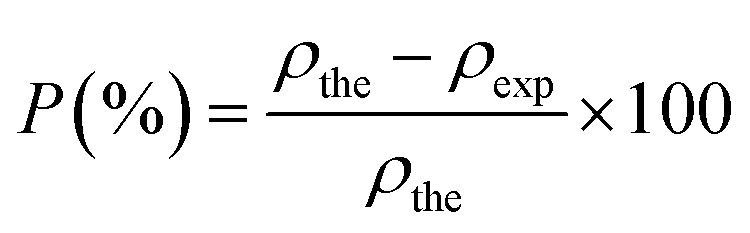


The determined *ρ*_X-ray_, *ρ*_exp_ and *P* are summarized in Table 1.

Assuming spherical particles, the specific surface area (*S*) was estimated using the following relation:^44^8
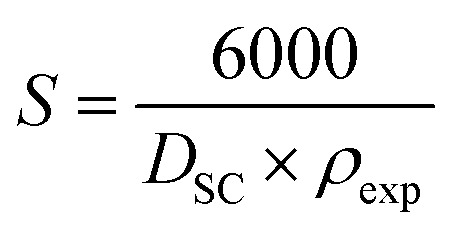
where *D*_W−H_is the crystallite size determined from the XRD analysis and *ρ*_exp_is the experimental density. The reduction in crystallite size with increasing Sr content results in an increase in the effective surface area, which is particularly beneficial for gas adsorption and sensing processes.

The dislocation density (*δ*), which reflects the concentration of crystalline defects within the material, was calculated using the following expression:^45^9
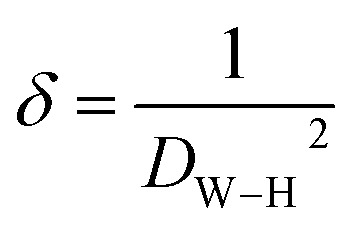


The substitution of Ba^2+^ by Sr^2+^ modifies the crystal lattice due to the smaller ionic radius of Sr^2+^. As summarized in Table 1, increasing the Sr content leads to a gradual decrease in the lattice parameter and crystallite size, accompanied by an increase in microstrain, surface area and dislocation density. The obtained strain values (1.34 × 10^−3^ − 3.15 × 10^−3^) are consistent with those reported for spinel ferrites,^46–48^ confirming the presence of significant crystalline distortions and defect generation within the spinel structure.

These defects, including lattice distortions, cation disorder, and oxygen-vacancy-related imperfections, strongly influence both charge transport and surface reactivity.^49^ The incorporation of smaller Sr^2+^ ions promotes the formation of oxygen vacancies, which act as donor defects in n-type ferrites and enhance the adsorption of oxygen species on the material surface. In particular, Sr incorporation may modify the mobility of oxygen vacancies as well as the nature of adsorbed oxygen species, thereby influencing surface reactivity and the gas adsorption–desorption process. The adsorbed oxygen species capture electrons from the conduction band, forming an electron depletion layer and increasing the electrical resistance. Upon exposure to reducing gases such as ethanol and acetone, the surface oxygen species react with the gas molecules and release electrons back to the conduction band, resulting in a decrease in resistance.

### 3.2 Morphological study

**Fig. 3:**
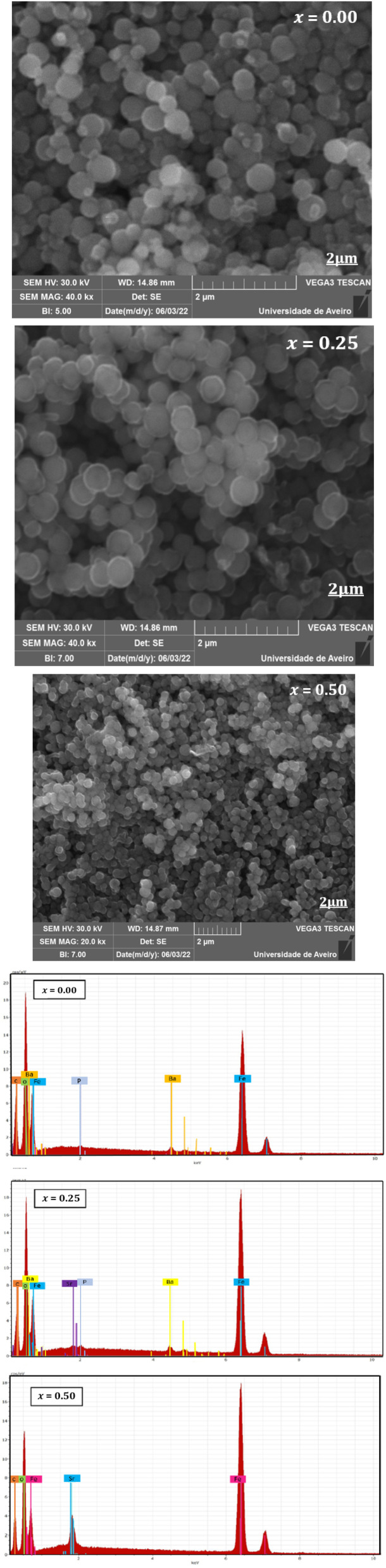
(a) SEM image and (b) EDS spectrum showing the elemental composition of the Ba_0.5−*x*_Sr_*x*_Fe_2.5_O_4_ (*x* = 0.00; 0.25 and 0.50) compound synthesized *via* the solvothermal method.

The morphological characteristics of the Ba_0.5−*x*_Sr_*x*_Fe_2.5_O_4_ nanoparticles were investigated using scanning electron microscopy (SEM) (Fig. 3(a)), a powerful technique for analyzing surface morphology and microstructural features. The SEM micrographs reveal nanometric particles that are strongly agglomerated and uniformly distributed over the entire surface, exhibiting a relatively homogeneous quasi-spherical morphology. This agglomerated and porous microstructure is particularly advantageous for gas-sensing applications, as it enhances gas diffusion and increases the number of active adsorption sites. The degree of agglomeration estimated using the grain-to-crystallite size ratio (*D*_MEB_/*D*_DRX_), ranges from 45.86 to 41.52 and 39.05 for *x* = 0.00, 0.25, and 0.50, respectively. These relatively high values indicate that each grain consists of multiple nanocrystallites, confirming a polycrystalline and strongly agglomerated nanostructure. The variation of this ratio with Sr content suggests modifications in crystallite assembly, grain growth behavior, and packing density within the microstructure.

Furthermore, Energy Dispersive X-ray Spectroscopy (EDS) was employed to confirm the elemental composition of the prepared ceramics. The EDS spectra (Fig. 3(b)) of all three compounds clearly reveal the presence of the characteristic peaks of Fe and O. For the sample with *x* = 0.00, the characteristic peak of Ba is detected. In the compound with *x* = 0.25, the characteristic peaks of both Sr and Ba are observed, indicating a partial substitution of Ba by Sr. When the substitution level reaches *x* = 0.50, only the Sr peak is detected, confirming the complete substitution of Ba by Sr in the studied structure.

In addition, the presence of all expected elements (Ba, Sr, Fe, and O) confirms the effective incorporation of the constituent elements into the synthesized materials, with no significant elemental loss during the synthesis process. The detected carbon (C) signal in the EDS spectra is attributed to the carbon adhesive tape used during sample preparation prior to analysis, while the weak phosphorus (P) signal is likely due to residues from the beaker coating used during the solvothermal synthesis process.

### 3.3 Detection performance of acetone

**Fig. 4:**
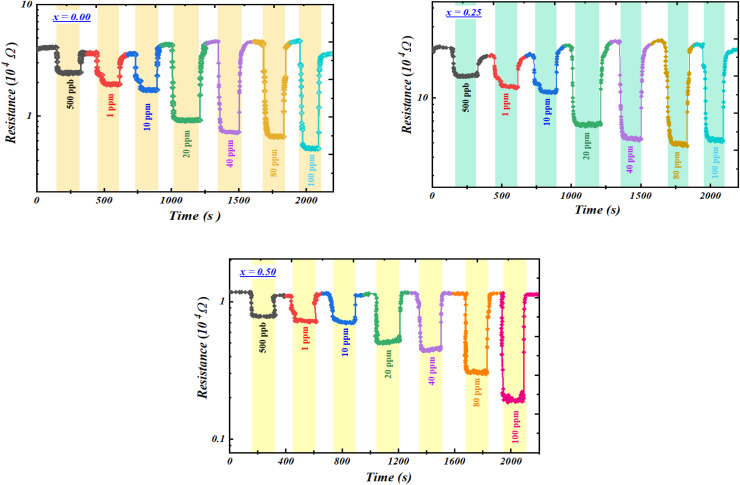
Resistance variation curves as a function of time for the compounds Ba_0.5−*x*_Sr_*x*_Fe_2.5_O_4_ compounds (*x* = 0.00, 0.25, and 0.50),measured at the optimal operating temperature (*T*_F_ = 200 °C) under different acetone concentrations.

The gas-sensing performance of the compounds toward acetone gas at different concentrations, investigated over the temperature range of 100–340 °C, reveals a decrease in resistance characteristic of n-type semiconductors (Fig. 4).^50^ This behavior is attributed to oxygen desorption and the catalytic reaction of acetone with Fe–O sites, producing CO_2_ and H_2_O along with the release of electrons into the crystal lattice.

**Fig. 5:**
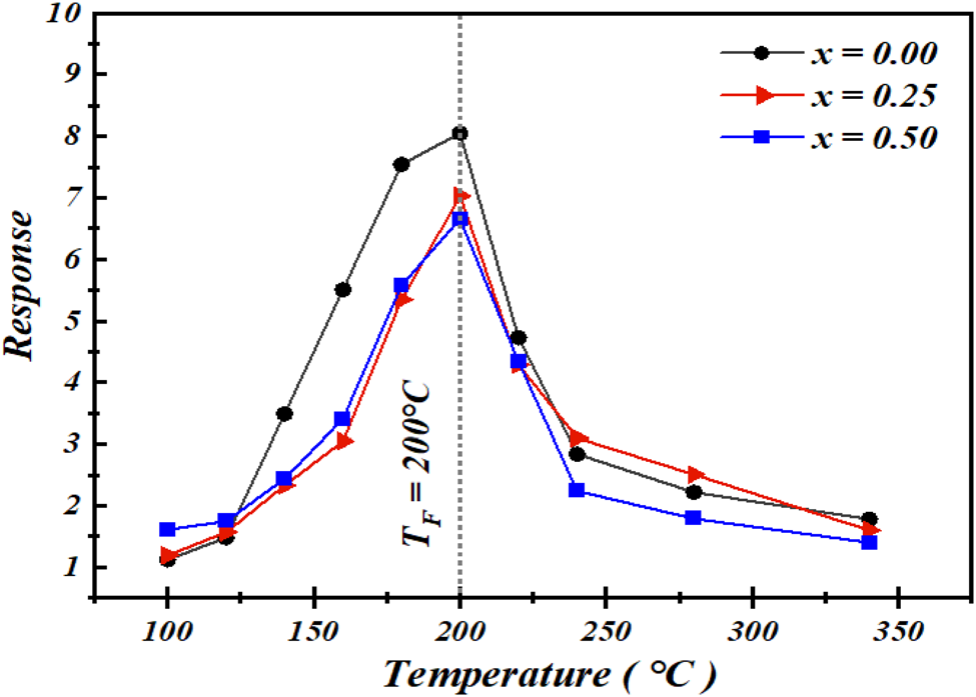
Response variation as a function of temperature for the compounds under 100 ppm acetone concentration.

Fig. 5 shows the relationship between operating temperature and gas response. It can be observed that, as the temperature increases, the gas response initially rises, reaches a maximum value at 200 °C, and then gradually decreases, exhibiting a “volcano-shaped” trend. This “increase–maximum–decrease” behavior can be explained as follows: at low temperatures, gas molecules are not sufficiently activated to overcome the activation energy barrier and react with the adsorbed oxygen species on the surface, resulting in a relatively low response. With increasing temperature, the enhanced reaction activity and the progressive conversion of adsorbed oxygen species (O_2_(ads) → O_2_^−^(ads) → O^−^(ads) → O^2−^(ads)) strongly contribute to the improvement of the response. At excessively high temperatures, gas adsorption becomes more difficult, reducing the effective utilization of the sensing material, which leads to a decrease in gas response.

It should be emphasized that increasing the substitution rate of barium with strontium (x) does not lead to a significant improvement in the gas response, although all three compositions exhibit high performance. As illustrated in Fig. 5, the optimal operating temperature (*T*_F_) is 200 °C for all the studied compositions.

The operating temperature reported here is significantly lower than that of many other materials commonly used for detecting the same gas, which represents a clear advantage for practical applications. For instance, Co_3_O^−^_4_ based sensors generally operate at temperatures between 300 °C^31^ and 380 °C,^39^ while *C*o_3_O_4_/NiFe_2_O_4_ composites require up to 239 °C.^51^ Likewise, WO_3_ operates at 300 °C for a concentration of 100 ppm,^52^ whereas ZnFe_2_O_4_ requires 275 °C under the same conditions.^53^

Moreover, we observe that the response values of our three compounds exceed those reported in the literature for a concentration of 100 ppm acetone (Table 2).

**Table 2:** Comparison between the operating temperature and response values of our Ba_0.5−*x*_Sr_*x*_Fe_2.5_O_4_ (*x* = 0.00; 0.25 and 0.50) compounds and the values reported for other sensors used for ethanol and acetone detection

Gas	Material	*T* (°C)	*C* (ppm)	*S* (*R*_a_/*R*_g_)	Ref.
Acetone	NiFe_2_O_4_	250	10/20	1.7/2.5	31 and 32
	MgFe_2_O_4_	420	100	1.82	31
	Co_3_O_4_	380	100	1.24	30
	ZnM_2_O_4_	206/250/270	5/10/50	2.6/1.36/4.2	33–35
	ZnO/ZnFe_2_O_4_	280	50/100	5.2/5.7	36
	Co_3_O_4_/NiFe_2_O_4_	239	100	3.09	27
	ZnFe_2_O_4_	275	100	6.5	29
	BiFeO_3_	220	100	5.5	37
	WO_3_	300	100	4.8	28
	MFe_2_O_4_	250	100	0.5	47
Ethanol	CoFe_2_O_4_	400	100	4.5	48
	Co_3_O_4_	300	100	1.43	17
	BiFeO_3_	260	20	1.9	49
	Au@Nio-nanoparticules	250	100	2.54	50
	WO_3_ nanosheets	300	100	4.8	28

**Fig. 6:**
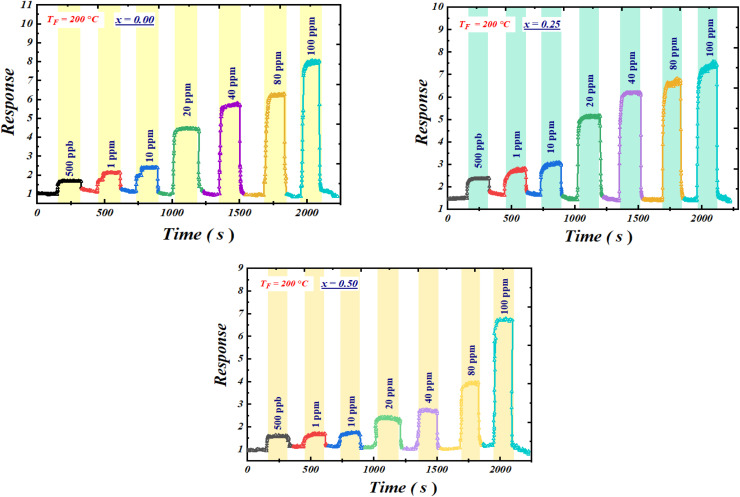
Time-dependent response curves for various acetone gas concentrations, measured at the optimal operating temperature TF = 200 °C.

**Fig. 7:**
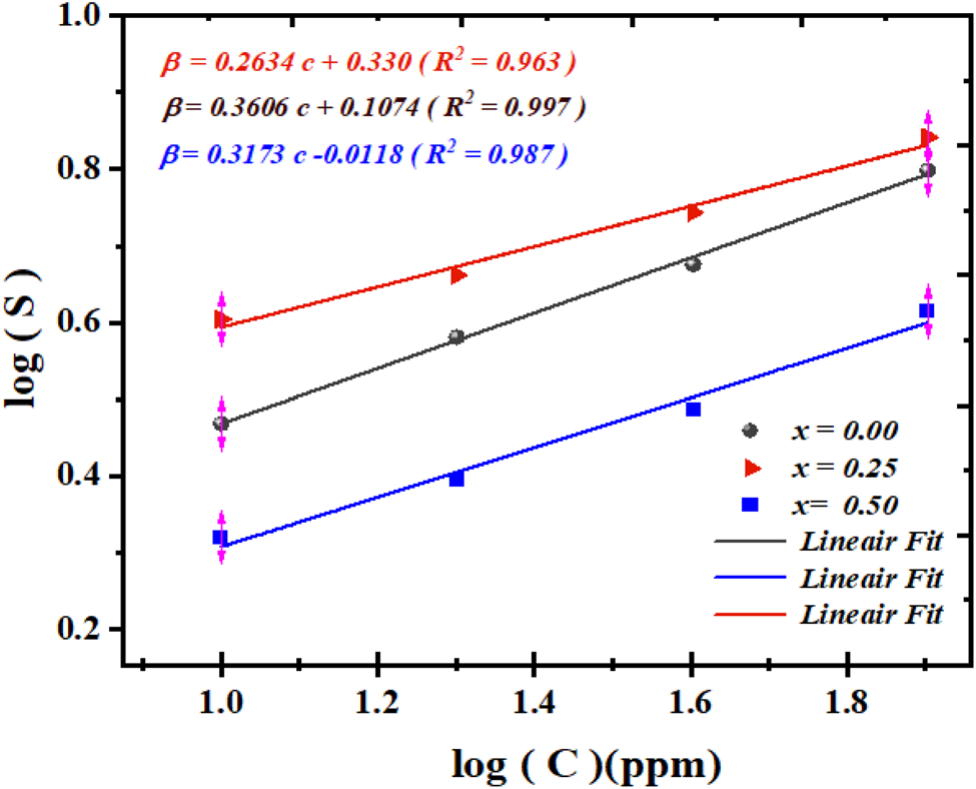
Calibration curves (log(*S*) *vs.* log(*C*)) for Ba_0.5−*x*_Sr_*x*_Fe_2.5_O_4_ (*x* = 0.00, 0.25, and 0.50) compounds, measured at their operating temperature of 200 °C under different acetone gas concentrations.

For instance, Shouli Bai^54^ reported a response value of 1.24 for Co_3_O_4_, a material widely recognized for acetone detection. Similarly, Ashok B. Gadkari^55^ observed that an iron–magnesium-based compound exhibited a response of 1.82 at 100 ppm of acetone. In addition, NiFe_2_O_4_ exhibited responses of 1.7, and 2.5 at 10, and 20 ppm, respectively.^55,56^ ZnMn_2_O_4_ demonstrated responses of 2.6, 1.36, and 4.2 at 5, 10, and 50 ppm, respectively.^57–59^ ZnO/ZnFe_2_O_4_ composites revealed higher responses of 5.2 and 5.7 at 50 and 100 ppm,^60^ while Co_3_O_4_/NiFe_2_O_4_ nanostructures showed a response of 3.09 at 100 ppm.^51^ Pure ZnFe_2_O_4_ reached a response of 6.5 at 100 ppm,^53^ and BiFeO_3_ exhibited a response of 5.5 under similar conditions.^61^ Finally, WO_3_ showed a response of 4.8 at 100 ppm of acetone.^52^ After determining the operating temperature, we examined the variation of the response (*S*) as a function of time for different acetone gas concentrations (Fig. 6). The obtained *S* values for each concentration are shown in Fig. 7.

The analysis of these results reveals that the response of the Ba_0.5−*x*_Sr_*x*_Fe_2.5_O_4_ compounds (*x* = 0.00, 0.25 and 0.50) gradually increases with increasing acetone concentration. Moreover, it remains difficult to draw a precise conclusion regarding the effect of Ba substitution by Sr, as the variation in response does not exceed 25%. Thus, the introduction of Sr does not appear to provide a significant improvement in response. Nevertheless, it should be emphasized that the response values obtained for acetone detection are sufficiently high to be considered very satisfactory for practical applications, such as air quality monitoring, industrial safety, medical diagnostics, or food quality control.

The limit of detection (LOD) is a key parameter for evaluating the sensitivity of a gas sensor, corresponding to the lowest concentration that can be reliably detected. It is determined from the calibration curve, which relates the sensor response to the gas concentration according to a power law:^62^

10 *S* = *kC*^*α*^

where *S* is the sensor response, *C* the gas concentration, *k* a sensor-specific constant, and *α* the slope obtained from the plot of log(*S*) *versus* log(*C*)^62^ (Fig. 7).

The LOD is defined as the minimum concentration at which the signal-to-noise ratio reaches a given threshold:^63^11
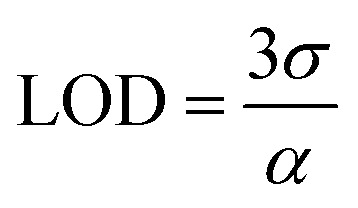
with *σ* representing the standard deviation of the background noise.

For the *x* = 0.00 sample, the linear fit yields a slope of 0.263, an intercept of 0.330, and a correlation coefficient of *R*^2^ = 0.963. For the *x* = 0.25 sample, the corresponding values are 0.360, 0.1074, and *R*^2^ = 0.997. Finally, for the *x* = 0.50 sample, a slope of 0.317, an intercept of −0.011, and *R*^2^ = 0.987 are obtained.

In comparison with other reported acetone sensors, the (LOD) values of our three compounds are inferior to those found in the literature (Table 3). For example, ZnFe_2_O_4_ and ZnO/ZnFe_2_O_4_ − ZnO exhibit LODs of 0.8 and 0.5 ppm, respectively,^64^ whereas SnO_2_ shows a much higher value of 54 ppm.^30^ Even lower limits have been reported for ZnO/ZnFe_2_O_4_ (0.05 ppm)^65^ and ZnCo_2_O_4_ (0.5 ppm).^66^ Similarly, NiFe_2_O_4_ presents an LOD of 1 ppm,^67^ while α−Fe_2_O_3_/NiFe_2_O_4_ composites reach 10 ppm.^68^ These comparisons suggest that although our three compounds exhibit inferior LOD values, they remain effective candidates for low-level acetone detection thanks to their good linearity and overall sensing performance.

**Table 3:** Comparison of the detection limit (LOD) values of our Ba_0.5−*x*_Sr_*x*_Fe_2.5_O_4_ (*x* = 0.00, 0.25 and 0.50) compounds with those reported for other sensors used for ethanol and acetone detection

Gas	Material	LOD (ppm)	Reference
Acetone	ZnFe_2_O_4_	0.8	40
	ZnO/ZnFe_2_O_4_–ZnO	0.5	40
	SnO_2_	54	16
	ZnO/ZnFe_2_O_4_	0.05	41
	ZnCo_2_O_4_	0.5	42
	NiFe_2_O_4_	1	43
	α−Fe_2_O_3_/NiFe_2_O_4_	10	44
	Ba_0.5_Fe_2.5_O_4_	0.022	**This work**
	Ba_0.25_Sr_0.25_Fe_2.5_O_4_	0.014	**This work**
	Sr_0.5_Fe_2.5_O_4_	0.021	**This work**
Ethanol	Co_3_O_4_	20	51
	ZnO	1	54
	SnO_2_	5	52
	Au/SnO_2_/ZnO	10	53
	Ba_0.5_Fe_2.5_O_4_	0.05	**This work**
	Ba_0.25_Sr_0.25_Fe_2.5_O_4_	0.016	**This work**
	Sr_0.5_Fe_2.5_O_4_	0.018	**This work**

**Fig. 8:**
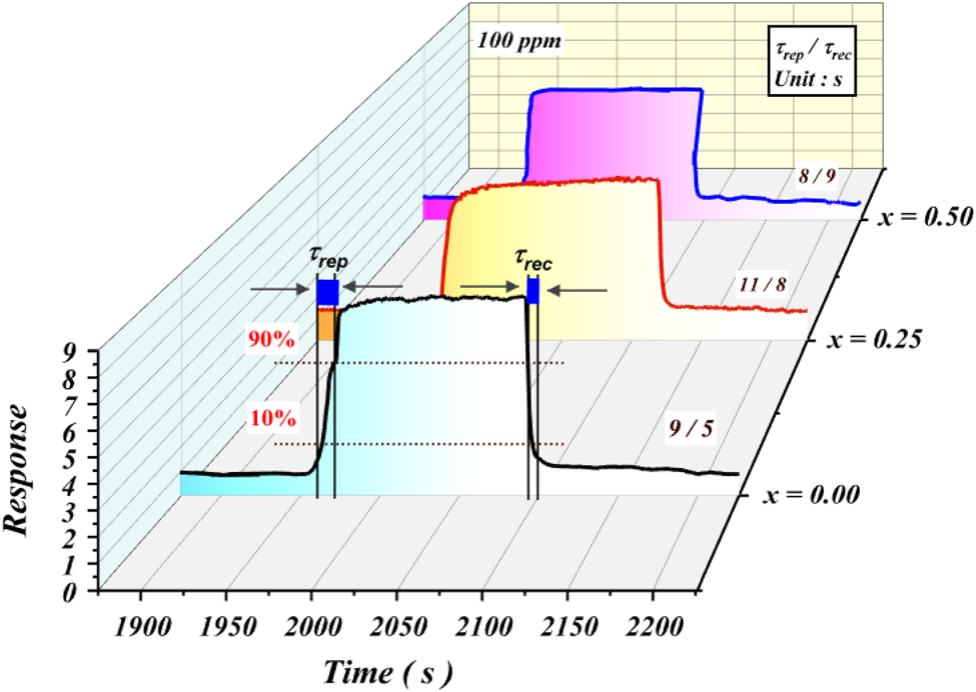
Examples of response time (*τ*_rep_) and recovery time (*τ*_rec_) determination for the compounds exposed to 100 ppm acetone at an operating temperature of 200 °C.

The response and recovery times of the Ba_0.5−*x*_Sr_*x*_ Fe_2.5_O_4_ (*x* = 0.00, 0.25 and 0.50) sensors, exposed to 100 ppm of acetone at 200 °C, were calculated and found to be remarkably short. These reduced times are attributed to the rapid adsorption and desorption reactions occurring on the sensor surface.^69^Fig. 8 illustrates examples of the determination of the response time (*τ*_rep_) and recovery time (*τ*_rec_) for the three compounds at an acetone concentration of 100 ppm. These values were calculated at the operating temperature *T* = 200 °C. As shown in Table 4, these relatively short times, with an average below 12 s, are significantly shorter than those reported in the literature for other sensors used to detect the same gas. For instance, the compound MgFe_2_O_4_ exhibits a response time of *τ*_rep_ = 180 s and a recovery time of *τ*_rec_ = 360 s at 100 ppm acetone.^70^ Similarly, Co_3_O_4_ shows response and recovery times of 48 s and 35 s, respectively, in the presence of 100 ppm acetone.^54^

**Table 4:** Response time (*τ*_rep_) and recovery time (*τ*_rec_) of our compounds at different acetone gas concentrations

*C* (pp*m*)	*x* = 0.00		*x* = 0.25		*x* = 0.50	
	*τ*_rep_ (*s*)	*τ*_rec_ (*s*)	*τ*_rep_ (*s*)	*τ*_rec_ (*s*)	*τ*_rep_ (*s*)	*τ*_rec_ (*s*)
0.001	10	8	8	6	8	5
0.01	9	9	9	7	11	2
0.1	11	11	11	7	10	5
0.5	10	8	7	7	7	4
1	12	9	9	6	9	5
5	7	9	11	8	8	9
10	11	11	8	7	12	9
20	7	8	10	8	9	8
50	9	10	8	10	11	9
80	8	11	11	11	8	6
100	9	5	11	8	8	9

### 3.4 Detection performance of ethanol

**Fig. 9:**
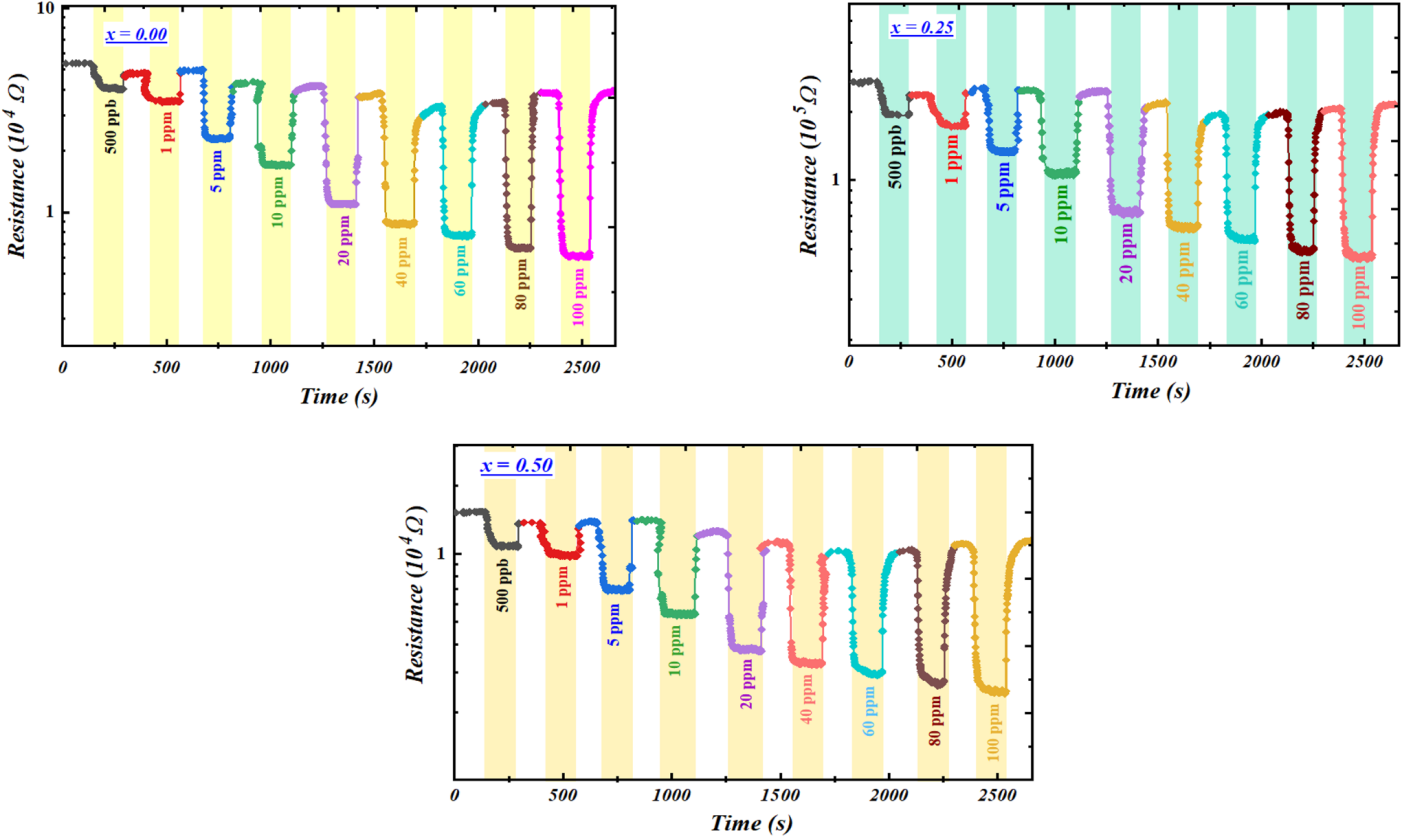
Resistance variation curves as a function of time for the compounds Ba_0.5−*x*_Sr_*x*_Fe_2.5_O_4_ compounds (*x* = 0.00, 0.25, and 0.50), measured at the optimal operating temperature (*T*_F_ = 200 °C) under different ethanol concentrations.

After characterizing our sensors based on Ba_0.5−*x*_Sr_*x*_Fe_2.5_O_4_ (*x* = 0.00, 0.25 and 0.50) in the presence of acetone, we then investigated their behavior toward ethanol gas. For this analysis, we applied the same experimental methodology as that used for acetone. As previously mentioned, the first step was to determine the optimal operating temperature of our compounds in the presence of ethanol. To this end, the three compounds were exposed to different concentrations of ethanol over a temperature range of 100 to 340 °C (Figure.9). The results indicate that the three compounds Ba_0.5−*x*_Sr_*x*_Fe_2.5_O_4_ (*x* = 0.00, 0.25 and 0.50) behave as n-type semiconductors. After the gas is removed, they recover their initial resistance, confirming the stability and reversibility of the materials. Moreover, the resistance curves show significant variations upon exposure to ethanol, even at very low concentrations, demonstrating high sensitivity. These findings not only highlight the effectiveness of our compounds in detecting ethanol but also confirm their potential for applications in low-concentration gas sensing.

The responses of the three compounds at different temperatures were determined, and the results are presented in Table 5. It can be observed that the response varies with temperature for all compounds, reaching a maximum at an optimal value, defined as the operating temperature. Notably, increasing the substitution rate (*x*) of barium with strontium does not lead to a significant improvement in the response; however, the response values for all three compounds remain considerably high. The data also show that the three compounds share the same optimal operating temperature of 200 °C. This value is significantly lower than those reported for other materials used for ethanol detection (Table 2), which represents a clear advantage for practical applications. Indeed, many metal oxides reported in the literature exhibit performance that is strongly dependent on both the operating temperature and the gas concentration.

**Table 5:** Response (*S*) values determined for an ethanol concentration of 100 ppm at different temperatures for the Ba_0.5−*x*_Sr_*x*_Fe_2.5_O_4_ (*x* = 0.00, 0.25 and 0.50) compounds

*T* (°C)	*S* (*R*_a_/*R*_g_)			*T* (°C)	*S* (*R*_a_/*R*_g_)		
	*x* = 0.00	*x* = 0.25	*x* = 0.50		*x* = 0.00	*x* = 0.25	*x* = 0.50
100	1.00	1.91	1.09	200	**8.75**	**6.47**	**7.76**
120	1.22	2.26	2.31	220	3.33	2.89	2.73
140	2.33	3.00	4.08	240	2.62	1.67	1.62
160	5.55	4.92	4.90	280	2.15	1.54	1.43
180	8.21	5.15	6.04	340	1.71	1.17	1.24

For example, MFe_2_O_4_(M = Zn, Cu, Ni and Co) exhibits a response of 0.5 for 100 ppm at 250 °C,^71^ while CoFe_2_O_4_ reaches 4.5 but at a higher temperature of 400 °C.^72^ Co_3_O_4_ shows a relatively low response of 1.43 at 300 °C for 100 ppm,^31^ whereas BiFeO_3_ can detect lower concentrations (20 ppm) with a response of 1.9 at 260 °C.^73^ Finally, among composite nanostructures, Au@NiO nanoparticles display a response of 2.54 at 250 °C for 100 ppm,^74^ while WO_3_ nanosheets reach a higher response of 4.8 at 300 °C for the same concentration.^52^

**Fig. 10:**
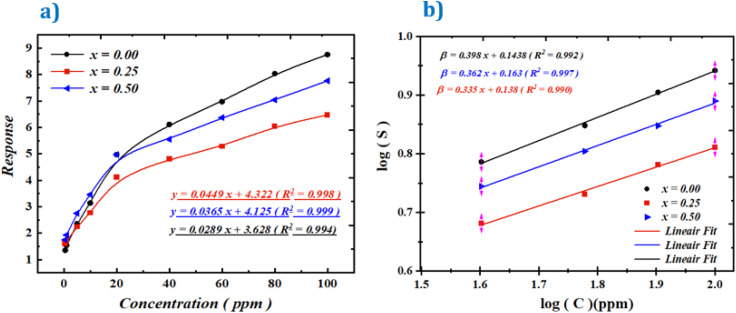
(a) Response variation of the sensors at different ethanol concentrations at 200 °C. (b) Calibration curves (log(response) *vs.* log(*C*)) of the Ba_0.5−*x*_Sr_*x*_Fe_2.5_O_4_ compounds (*x* = 0.00, 0.25, and 0.50) measured at 200 °C.

After determining the operating temperature, we investigated the time-dependent response of the samples for different ethanol concentrations at 200 °C. As previously observed for acetone, the response clearly increases with increasing ethanol concentration, as illustrated in Fig. 10(a).

This figure shows a linear variation at high ethanol concentrations for each of the three compounds. However, it remains difficult to draw a precise conclusion regarding the effect of substituting Ba with Sr, as the response fluctuation does not exceed 25%. Nevertheless, it can be noted that the response values obtained for ethanol are sufficiently high to be considered very satisfactory for applications in ethanol gas detection, as previously discussed.

The results demonstrate that the sensors exhibit low detection limits (LOD), confirming their suitability for trace ethanol detection in applications such as environmental monitoring, industrial safety, and medical diagnostics. As shown in Fig. 10(b) and Table 3, the response curves display excellent linearity (*R*^2^ > 0.99 for all compositions), with the *x* = 0.25 substituted material showing the best performance.

Compared with other ethanol sensors reported in the literature-such as Co_3_O_4_ (20 ppm)^75^,SnO_2_ (5 ppm),^76^ Au/SnO_2_/ZnO (10 ppm),^77^ and ZnO (1 ppm)^78^ our compounds remain competitive, combining good sensitivity with strong linearity.

The response (*τ*_rep_) and recovery (*τ*_rec_) times of the three compounds at different ethanol concentrations are relatively short, with an average of less than 10 s, which is significantly lower than those reported for other ethanol gas sensors in the literature.

## 4 Mechanism of Vocs detection

According to the well-established space-charge layer model for n-type metal oxide gas sensors, the sensing performance mainly depends on the modulation of the thickness of this layer.^79^ When the sensors are exposed to ambient air, oxygen molecules rapidly adsorb onto the surface of the sensitive materials and are converted into more reactive chemisorbed oxygen species (O_2_^−^, O^−^ and O^2−^) by extracting electrons from the material. This electron transfer leads to the formation of an electron-depleted layer at the surface, resulting in an increase in the sensor's resistance.

When a reducing gas such as acetone or ethanol comes into contact with the sensor, the redox reaction between the gas and the adsorbed oxygen species dominates the surface chemical process. The electrons trapped in the oxygen ions are released into the conduction band of the material, reducing the electron-depleted layer and resulting in a significant sensing response.^80^ These processes can be represented by the following chemical reactions:^81^

12 O_2(gaz)_ ↔ O_2(ads)_

13 O_2(ads)_ + e^−^ ↔ O_2(ads)_^−^

14 O_2(ads)_^−^ + e^−^ ↔ 2O_(ads)_^−^

15 O_(ads)_^−^ + e^−^ ↔ O_(ads)_^2−^

The chemical reaction of ethanol at an operating temperature of 200 °C can be described as follows:^82^

16 C_2_H_5_OH (gaz) + 6O^−^ (ads) → 2CO_2_ + 3H_2_O + 6e^−^

For acetone, the operating temperature of 200 °C can be described Vas follows:^83^

17 CH_3_COCH_3_ (gaz ) + 8O^−^ (ads) →3CO_2_ + 3H_2_O + 8e^−^

The electrons released during these reactions increase the concentration of free electrons in the conduction band, thereby decreasing the sensor's resistance. The correlation between the gas concentration and the change in resistance allows for the measurement of the target gas.^84^

On the other hand, nanoferrite nanoparticles can act as effective sites for dissociating oxygen molecules into more reactive radicals, a phenomenon known as chemical sensitization. As a result, the adsorption of oxygen species and surface reactions are significantly accelerated, leading to a notable increase in response speed. Therefore, it can be concluded that the significant enhancement of gas-sensing performance of the Ba_0.5−*x*_Sr_*x*_Fe_2.5_O_4_ compounds is due not only to their unique structural features, such as small size and high specific surface area, but also to the sensitization effect induced by the nanoparticles.

## 5 Conclusion

In this study, Ba_0.5−*x*_Sr_*x*_Fe_2.5_O_4_ nanopowders were successfully synthesized using the solvothermal method, yielding well-crystallized materials with a cubic structure and nanometric crystallite sizes. Increasing the strontium content leads to a reduction in lattice parameters, accompanied by a decrease in crystallite size and porosity.

Gas sensors fabricated from these metal oxides exhibited n-type semiconductor behavior over a wide temperature range and showed high sensitivity toward acetone and ethanol. Experimental results revealed strong sensor responses even at very low gas concentrations, with fast response and recovery times and an optimal operating temperature of 200 °C. The performance was similar across all compositions, indicating that increasing the strontium content does not significantly enhance the sensitivity or selectivity of the sensors for these gases.

The sensor response increases with gas concentration, reflecting more intense interactions between the gas molecules and adsorbed oxygen on the surface. The average response and recovery times, around 12 s, are notably shorter than those reported in the literature for similar sensors.

The improved sensing performance is attributed to increased lattice oxygen activity at the surface, the formation of oxygen vacancies, and enhanced gas adsorption capacity, which promote more effective interactions between gas molecules and active sites on the sensor surface. Furthermore, Ba_0.5−*x*_Sr_*x*_Fe_2.5_O_4_ nanoparticles proved to be sensitive, selective, and promising materials for efficient detection of acetone and ethanol. Their combination of optimal low-temperature performance, fast response and recovery times, and low detection limits opens new perspectives for the development of ferrite-based gas sensors.

## Conflicts of interest

There are no conflicts to declare.

## Data Availability

The data supporting this study are available upon request but not for sharing.
